# Occurrence of Neonatal Necrotizing Enterocolitis in Premature Neonates and Gut Microbiota: A Case–Control Prospective Multicenter Study

**DOI:** 10.3390/microorganisms11102457

**Published:** 2023-09-29

**Authors:** Julio Aires, Zehra Esra Ilhan, Lancelot Nicolas, Laurent Ferraris, Johanne Delannoy, Maxime Bredel, Anne Chauvire-Drouard, Frédéric Barbut, Jean-Christophe Rozé, Patricia Lepage, Marie-José Butel, ClosNEC Study Group

**Affiliations:** 1 Faculté de Pharmacie de Paris, Université Paris Cité, INSERM, UMR-S 1139 (3PHM), 75006 Paris, Francefrederic.barbut@aphp.fr (F.B.);; 2 FHU PREMA, Fighting Prematurity, 75014 Paris, France; 3 Micalis Institute, INRA, AgroParisTech, University Paris-Saclay, 91190 Paris, France; zehra-esra.ilhan@inrae.fr (Z.E.I.); maxime.bredel@inrae.fr (M.B.);; 4 Centre d’Investigation Clinique CIC 1413, INSERM, CHU de Nantes, 44093 Nantes, France; anne.drouard@chu-nantes.fr; 5 Department of Neonatal Medicine, University Hospital of Nantes, 44093 Nantes, France; jcroze.chu@gmail.com

**Keywords:** necrotizing enterocolitis, NEC, multicenter study, gut microbiota, UPEC, uropathogenic *Escherichia coli*, *Clostridium butyricum*, *Clostridium neonatale*, antimicrobial susceptibility

## Abstract

Background: Necrotizing enterocolitis (NEC) is still one of the leading causes of neonatal death. The present study reports the data from a French case–control prospective multicenter study. Methods: A total of 146 preterm neonates (PNs) with or without NEC were included. Bacterial 16S rRNA gene sequencing was performed on stool samples (n = 103). Specific culture media were used to isolate *Escherichia coli*, *Clostridium butyricum*, and *Clostridium neonatale*, and strains were phenotypically characterized. Results: The gut microbiota of PNs was dominated by Firmicutes and Proteobacteria, and five enterotypes were identified. The microbiota composition was similar between NEC cases and PN controls. However, differences were observed in the relative abundance of *Lactobacillus* genus, which was significantly lower in the NEC group, whereas that of the *Clostridium* cluster III was significantly higher (*p* < 0.05). Within enterotypes, several phylotypes were significantly more abundant in NEC cases (*p* < 0.05). Regarding perinatal factors, a statistical association was found between the gut microbiota and cesarean delivery and antifungal therapy. In NEC cases and PN controls, the carriage rates and virulence genes of uropathogenic *E. coli* were equivalent based on culture. No correlation was found between *E. coli*, *C. butyricum*, and *C. neonatale* carriages, beta-lactam resistance, and antibiotic treatment. Conclusions: At disease onset, our data support a microbiota dysbiosis between NEC and control infants at the genus level. In addition, it provides valuable information on bacterial antimicrobial susceptibility.

## 1. Introduction

Necrotizing enterocolitis (NEC) represents one of the most severe and life-threatening gastrointestinal diseases in the neonatal intensive care unit (NICU). With an often unpredictable and sudden onset, NEC remains one of the leading cause of neonatal death and is responsible for significant morbidity and mortality [1]. The incidence of NEC ranges from 2% to 7% in infants born at a gestational age of less than 32 weeks (wks) and from 5% to 22% in infants with a birth weight less than 1000 g, depending on geographic area and time of report [2]. Risk factors for NEC include preterm birth, formula feeding, prolonged use of antibiotics, and gut microbiota dysbiosis [3]. To date, biomarkers or scoring systems have not been implemented due to their poor sensitivity, specificity, or predictive value for diagnosing or preventing NEC [3,4].

The pathogenesis of NEC is caused by intestinal immaturity, microbial dysbiosis, enteral feeding, and inflammation, resulting in excessive intestinal cell death, barrier dysfunction, and a subsequent inflammatory response [3]. In particular, gut bacteria play a role in triggering local inflammation, and gut microbiota dysbiosis is associated with increased susceptibility to NEC [4,5,6]. The fact that NEC could not be reproduced in germ-free animals strongly supports the role of bacteria in the development of the disease [6]. The gut microbiota of preterm neonates (PNs) with and without NEC has been studied before and during the disease using culture and culture-independent methods, leading to different conclusions about the involvement of the gut microbiota in NEC [7,8,9,10,11,12,13,14,15,16,17]. However, NEC occurs during the period of gut microbiota diversification and transition from facultative to strict anaerobes [4,5]. To date, no single microorganism has been conclusively associated with NEC. However, several studies have converged on a risk-associated community state consisting of an overrepresentation of bacteria all belonging to the gut commensal microbiota such as the *Enterobacteriaceae* family and certain *Clostridium* species [11,14,15,16,18,19,20,21,22]. In particular, uropathogenic *Escherichia coli* (UPEC), *Clostridium butyricum*, and *Clostridium neaonatale* intestinal colonization have been associated with NEC [11,14,22,23,24].

In this study, we present data from the nationwide prospective multicenter matched case–control study ClosNEC. Our aim was to compare the diversity and composition of the gut microbiota of French PNs with and without NEC. To achieve this, we used culture and metagenomics methods targeting the bacterial 16S rRNA gene. Second, we used this study to culture strains of *E. coli*, *C. butyricum*, and *C. neonatale*, species consistently associated with NEC and determine their antimicrobial susceptibility patterns. This was noteworthy because an association between the frequency of Enterobacteriaceae or *C. butyricum* colonization in NEC patients and antibiotic use has been proposed [23,24]. Third, as one study has suggested that gut colonization with UPEC is a risk factor for NEC [11], we examined the carriage of UPEC strains between NEC cases and controls and screened the strain for UPEC-specific virulence genes.

## 2. Materials and Methods

### 2.1. Study Design and Patients

**Figure 1:**
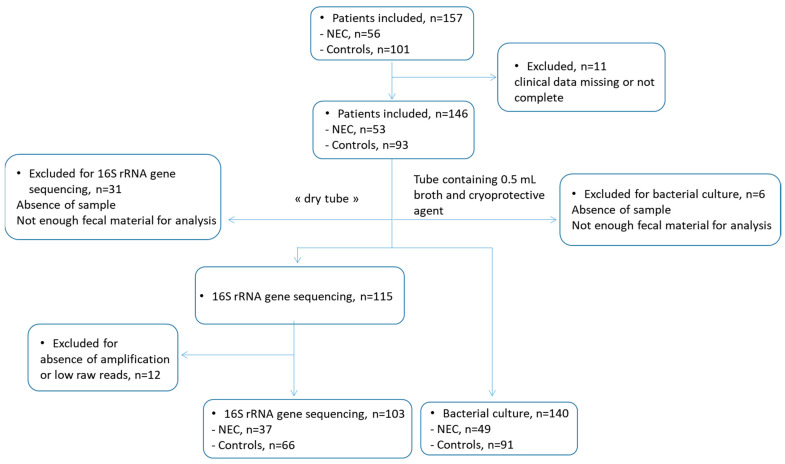
ClosNEC cohort flow chart.

PNs were recruited over a 16-month period (March 2015 to August 2016) in 20 French NICUs participating in the ClosNEC trial (NCT02444624). Approval was obtained from the National Data Protection Authority (Commission Nationale de l’Informatique et des Libertés, n° 915094) and the Consultative Committee on the Treatment of Information on Personal Health Data for Research Purposes (n° 15.055). Parents provided informed consent. All enrolled PNs were less than 32 weeks (wks) gestational age. NEC cases and control PNs were from the same NICU and were matched for gestational age, birth weight, feeding type, and mode of delivery. Each NEC case was matched with two controls. NEC was confirmed by the neonatal clinical team and defined as the presence of clinical evidence meeting the modified criteria for NEC Bell’s stage II (associated with radiologic pneumatosis intestinalis) or III (definitive intestinal necrosis seen at surgery or autopsy). Bell’s stages II and III corresponded to medical and surgical NEC, respectively [4]. The mean age of onset of NEC was 29 ± 20 days (d). PNs who were diagnosed with spontaneous intestinal perforation were excluded. No NEC outbreak was reported during the inclusion period; therefore, NEC cases were considered sporadic. A flow chart depicting the ClosNEC workflow is shown in Figure 1.

### 2.2. Fecal Samples

For NEC cases, stool samples were collected at the time of diagnosis (the first stool issued after the diagnosis). For neonates in the control group, stool samples were collected within one week of their matched NEC cases in the same NICU (mean 7.5 d). Stool samples were obtained at a mean gestational age of 29 ± 20 d for the NEC cases and 30 ± 21 d for the control PNs. Stools were collected from diapers, placed in 2 sterile tubes (with or without 0.5 mL brain-heart infusion broth with 15% glycerol as a cryoprotectant), and immediately frozen at −80 °C until DNA extraction for 16S rRNA gene sequencing and bacterial culture.

### 2.3. DNA Extraction, Sequencing, and Microbiota Analysis

Total DNA was extracted from 150 mg aliquots stored at −80 °C without reagents according to Lepage et al. [25]. Bacterial load was assessed by quantitative PCR of the Bacteria domain (F_Bact1369: CGGTGAATACGTTCCCGG and R_Prok1492: TACGGCTACCTTGTTACGACTT), as previously described [26]. Standard curves were generated by plotting threshold cycles (*C*_t_) versus bacterial count (CFU) expressed as CFU/g of fecal sample. Microbiota composition and diversity were analyzed by amplicon sequencing of the 16S rRNA V3–V4 region (V3fwd: TACGGRAGGCAGCAG; V4rev: TACCAGGGTATCTAAT) [27]. Raw reads were analyzed using the Quantitative Insights Into Microbial Ecology (QIIME 2) software package 2018.4 [28]. After primer and barcode trimming, sequences were filtered for quality (minimum length = 350-bp, minimum quality threshold = 20, and chimera removal) and clustered into de novo operational taxonomic units (OTUs) at a 97% similarity threshold using uclust. OTUs represented by fewer than 3 samples were removed from the OTU table. Samples that were amplified but resulted in less than 1000 reads were also removed. The most abundant member of each OTU was selected as a representative sequence and assigned to different taxonomic levels using the RDP naive Bayesian classifier and the RDP Seqmatch program [29].

### 2.4. E. coli, C. butyricum, and C. neonatale Strain Isolation and Identification

A total of 140 fecal samples were analyzed by culture (Figure 1). For *E. coli*, *C. butyricum*, and *C. neonatale* isolation stool samples were crushed in brain–heart infusion broth using an Ultra-Turrax T25 (Fisher-Bioblock, Illkirch, France), diluted in peptone water, and 10^−2^, 10^−4^ and 10^−6^ dilutions were spread on selective media using a WASP apparatus (Don Whithley Scientific, Bingley, UK), as previously described [30]; Drigalski agar (Bio-Rad, Marnes la Coquette, France) Gram-negative selective medium was used for *E. coli* isolation; and sulfite–polymyxin–milk selective agar medium was used for *C. butyricum* and *C. neonatale* isolation. The media were incubated at 37 °C for 24 h under aerobic conditions for *E. coli* or 48 h under anaerobic conditions (H_2_:CO_2_:N_2_, 10:10:80, *v*/*v*/*v*) in an anaerobic chamber (Don Whitley Scientific, Bingley, UK) for clostridia. Strains were conserved in brain–heart infusion medium containing 15% (*v*/*v*) glycerol at −80 °C. Subsequent bacterial cultures were performed under aerobic (*E. coli*) or anaerobic (*C. butyricum* and *C. neonatale*) conditions. Species identification was performed using matrix-assisted laser desorption ionization–time of flight mass spectrometry (MALDI-TOF MS) (Bruker Daltonics S.A., Wissembourg, France) and sequencing of the 16S rDNA gene, as previously described [31]. Briefly, for MALDI-TOF MS strain identification, colonies were deposited onto a target plate (Bruker Daltonics S.A.), were dried at room temperature, and 1 μL of saturated matrix solution containing HCCA (cyano-4-hydroxycinnamic acid in 50% acetonitrile and 2.5% trifluoroacetic acid) was added to each sample. For each strain, three independent replicate experiments were performed on a MALDI-TOF MS Microflex spectrometer (Bruker Daltonics S.A.), as previously reported [31]. Bacterial counts were expressed as log_10_ colony forming units (CFU)/g of feces (the count threshold was 3.3 log_10_ CFU/g of feces).

### 2.5. Phylogenetic and Virulence Characterization of E. coli Strains

Bacterial DNA from the isolates was extracted using the InstaGene™ Matrix kit, according to the manufacturer’s recommendations (Bio-Rad, Hercules, CA, USA). The phylogeny of the *E. coli* strains was determined by multiplex PCR based on amplification of the *arpA*, *chuA*, *yjaA*, *Tsp.E4*, and *TrpA* genes, as previously described [32]. Amplification by PCR of the capsular and virulence genes was performed as previously described [33,34]. PCR products were visualized by electrophoresis on 2% agarose gels prepared in 0.5× Tris-borate-EDTA buffer and visualized in Image Lab™ (Bio-Rad).

### 2.6. Antimicrobial Susceptibility

Antimicrobial susceptibility testing was performed by disk diffusion according to the EUCAST recommendations [35]. The antibiotics (bioMérieux, Marcy-l’Étoile, France) tested are listed in Tables S4 and S5. Minimal inhibitory concentrations (MICs) of tetracycline, clindamycin, and cefotaxime were determined using the Etest strips according to the manufacturer’s instructions (bioMérieux). Rapid detection of β-lactamase was performed using the nitrocefin disk test according to the manufacturer’s instructions (bioMérieux).

### 2.7. Statistical Analysis

Statistical analysis was performed using R software version 3.1.3 (http://www.R-project.org) (last accessed on 20 February 2023). Prior to calculating α-diversity, the 16S rRNA gene sequencing data set was rarefied at the level of 2500 reads. The calculated richness and α-diversity indices were the Chao 1 index, the number of observed OTUs, the Shannon, and the Simpson indices. Differences in diversity and relative abundances of taxa were tested using the Wilcoxon rank-sum test. Where appropriate, analyses were corrected for multiple testing (Benjamini-Hochberg procedure) [36]. The distance matrix for β-diversity analysis was computed using the vegdist and Bray–Curtis methods. Principal Component Analysis (PCA) and Principal Coordinate Analysis (PCoA) were computed on either compositional data or distance matrix data. Enterotypes were statistically defined as previously published by Arumugam et al. [37]. Two-sided Fisher’s exact, Mann–Whitney U, or Kruskal–Wallis tests were used to compare population characteristics, as appropriate, and the significance level was set at *p* < 0.05. Association analysis between gut microbial diversity and specific clinical variables was performed using Spearman’s rank correlation.

## 3. Results

### 3.1. Population Characteristics

Our population was homogenous with respect to clinical characteristics, including gestational age, birth weight, human milk intake, and mode of delivery (Table 1). With regard to antibiotics, early antibiotic therapy was defined as antibiotic administration initiated at or before 72 h of age. In the present study, early antibiotic therapy included the use of cefotaxime (n = 32, 39%), amoxicillin (n = 19, 23%), combination antibiotics (n = 18, 22%), aminoglycosides (n = 10, 12%), or other antimicrobials (n = 4, 5%). If a neonatal maternofetal or nosocomial infection was suspected, antibiotic treatment included vancomycin (n = 33, 34%), cefotaxime (n = 26, 28%), aminoglycosides (n = 12, 13%), or other antibiotics (n = 15, 16%). Regarding the time of fecal sampling, antibiotics were administered for a mean of 10 ± 9.6 d in the NEC cases and a mean of 19.5 ± 10.5 d in the PN control group. Statistically, NEC cases were treated significantly more often than controls for suspected maternofetal or nosocomial infections (Table 1). No statistical associations were observed with the most frequently used molecules (cefotaxime, amoxicillin, and vancomycin). Among mothers who received antenatal antibiotherapy, the treatments included the use of amoxicillin (n = 28, 62%), cefixime (n = 3, 7%), metronidazole (n = 3, 7%), or other antibiotics (n = 6, 14%). Per-partum antibiotic treatment consisted of amoxicillin (n = 34, 68%), cefotaxime (n = 5, 10%), or other drugs (n = 11, 22%). Statistically, there were no differences between the maternal antibiotic treatment and the two PN groups (Table 1).

Some PNs received antifungal treatment at a median age of 8 d, including fluconazole (n = 22, 52%), micafungin (n = 12, 29%), or other antifungals (n = 7, 17%). No statistical difference was observed between the NEC and control groups.

### 3.2. Gut Microbiota Analysis

**Figure 2:**
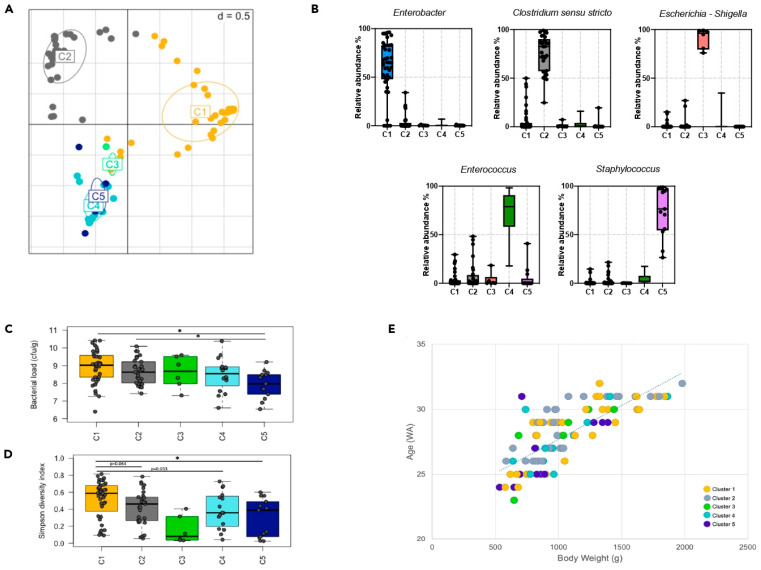
Specificities of the preterm gut microbiota. (**A**). Unsupervised cluster analysis based on gut microbiota composition described based on 97% sequence similarity and demonstrated by principal coordinate analysis. Microbiota can be stratified into five clusters (color coded). (**B**). Relative abundance of the major driving genera in each microbiota cluster. (**C**). Bacterial load expressed as CFU/g of stool samples based on qPCR analysis of the “All Bacteria” domain. (**D**). Bacterial diversity highlighted by the Simpson diversity index. (**E**). Cluster distribution as a function of age (in weeks of amenorrhea; WA) and body weight (in grams; g). * *p*-value < 0.05.

A total of 103 fecal samples (37 NEC, and 66 control PNs) were analyzed by 16S rRNA gene sequencing (Figure 1). The phyla Firmicutes and Proteobacteria were the most abundant taxa. The microbiota was dominated by Firmicutes (>50% relative abundance, average 91%) in 58 infants, by Proteobacteria (average 85%) in 42 infants, by Actinobacteria (average 54%) in two infants, and by both Firmicutes and Proteobacteria (49% each) in one infant. Based on the gut microbiota composition at the genus level, PNs were stratified into five enterotypes, namely, C1 to C5 (Figure 2A). These enterotypes were, respectively, driven by *Enterobacter* (n = 39), *Clostridium sensu stricto* (n = 29), *Escherichia* (n = 6), *Enterococcus* (n = 16), and *Staphylococcus* (n = 13) (Figure 2B). The average bacterial load was 4 × 10^8^ CFU/g of feces and was significantly lower in C5 (Figure 2C). The Simpson diversity index (Figure 2D) was highest in C1 (*p* < 0.05 when compared to C5 and *p* < 0.1 when compared to C2, C3, and C4). Although PNs from cluster C2 (enriched in *Clostridium sensu stricto*) were all older than 26 weeks, belonging to a particular clusters was not statistically related to gestational age or birth weight (Figure 2E). Gut microbial diversity and bacterial load were not correlated with either age or birth weight (Spearman r < 0.3).

### 3.3. Gut Microbiota in Preterm Neonates with or without NEC

**Figure 3:**
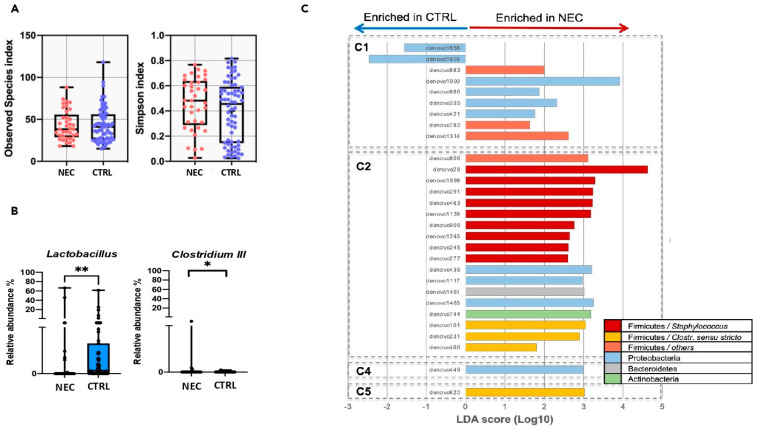
Comparison of the gut microbiota between the NEC cases and the control preterm neonates. (**A**). Richness (number of observed OTUs) and alpha-diversity (Simpson index) of the gut microbiota in NEC cases and control preterm neonates. No significant difference was found. (**B**). Bacterial genera are significantly different between the NEC cases and control preterm neonates. (**C**). Specific phylotypes (OTUs) significantly associated with the microbiota of NEC cases or controls based on LEfSe analysis (Linear discriminant analysis Effect Size). NEC: infants with necrotizing enterocolitis; CTRL: infants without NEC; C: Cluster; LDA: Linear discriminant analysis; Bacterial taxonomy is color coded as indicated; ** *p*-value < 0.001, * *p*-value < 0.05.

The distribution of NEC cases and controls did not differ between Firmicutes or Proteobacteria-dominated microbiota (Chi-square = 0.037, *p* = 0.846) nor among the five different enterotypes (Chi-square = 2.37, *p* = 0.668). Alpha diversity was equivalent between the NEC cases and controls and was highly variable in both groups (Figure 3A). Overall, the dominant microbiota composition did not differ between the NEC cases and controls. However, the relative abundance of the *Lactobacillus* genus was significantly lower, and that of the *Clostridium* cluster III was higher in the NEC cases compared to the controls (*p* < 0.05) (Figure 3B). The relative abundance of both genera was very low (median < 1% in both groups). When comparing NEC and controls within each enterotype, several phylotypes were significantly more abundant in NEC cases (Figure 3C). In the PNs of C1 (driven by *Enterobacter*), NEC-associated phylotypes included two OTUs related to *Klebsiella*, one related to *Coprococcus*, three related to *Enterobacter*, and one related to *Clostridium* XI (*C. bifermentans*). In the PNs of C2 (driven by *Clostridium sensu stricto*), 18 NEC-associated phylotypes were identified: nine OTUs related to *Staphylococcus*, three related to *Clostridium sensu stricto* (*C. neonatale* and one related to *C. perfringens*), two OTUs related to *Serratia,* and four OTUs related to *Dermabacter*, *Diaphorobacter*, *Prevotella*, and *Ruminococcus*.

### 3.4. Perinatal Factors and Gut Microbiota

**Figure 4:**
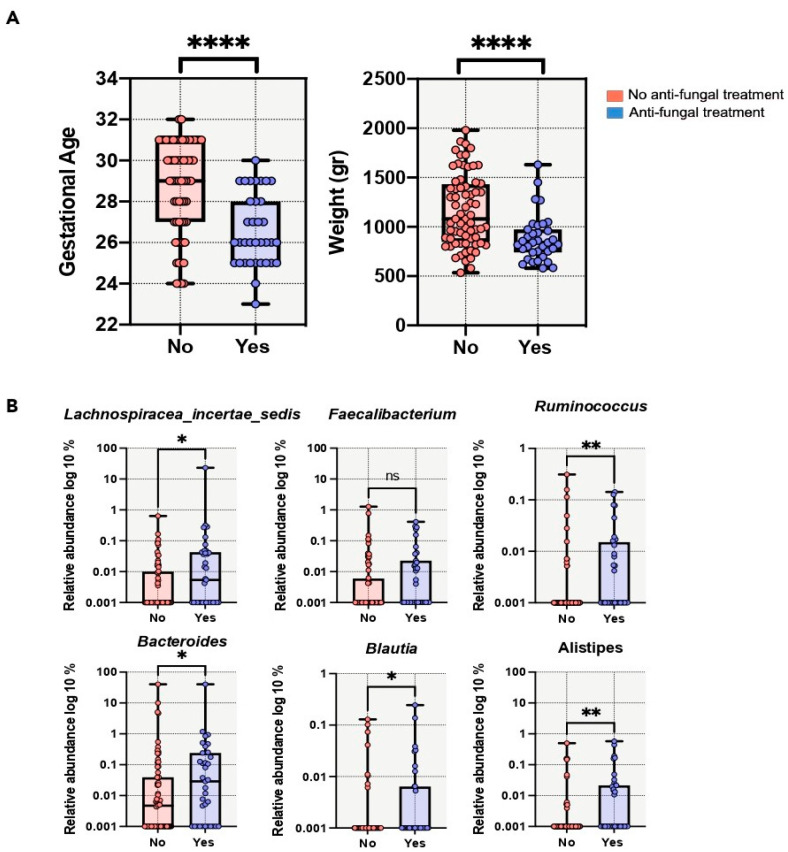
Impact of antifungal treatment on the preterm gut microbiota. (**A**). Antifungal treatments were significantly more frequent in preterm neonates with the lowest gestational age (in weeks) and birth weight (in grams). (**B**). Proportions of bacterial genera were significantly different between antifungal-treated and untreated PNs. **** *p*-value < 0.0001, ** *p*-value < 0.001, * *p*-value < 0.05; ns: non-significant; No: untreated group; Yes: Treated group.

With respect to our PN population, a statistical association was observed between cesarean delivery and antifungal use. Cesarean delivery was associated with a higher proportion of Firmicutes and a lower proportion of Proteobacteria (Supplementary Figure S1). Cesarean-born PNs had significantly higher proportions of *Clostridium cluster XI*, *Staphylococcus*, *Ruminococcus*, and *Corynebacterium* groups than those born vaginally. The use of antifungal treatment showed a significant association with low gestational age and low birth weight (Figure 4). There were also significant differences in the compositions of the gut microbiota (Figure 4A). *Bacteroides*, *Lachnospiraceae incertae sedis*, *Alistipes*, *Ruminococcus*, and *Blautia* had significantly higher relative abundances in PNs treated with antifungals (Figure 4B). On the other hand, the relative abundance of *Faecalibacterium* was slightly higher in the non-antifungal-treated group (*p* = 0.052).

No association between perinatal factors and gut microbiota was observed in NEC cases and controls.

### 3.5. E. coli Isolation, Characterization, and Antimicrobial Susceptibility Levels

A total of 21 *E. coli* strains were isolated from the fecal samples of NEC cases (n = 4, 19%) and from controls (n = 17, 81%). The mean level of strain colonization was similar between NEC cases and controls (7.9 ± 1.3 and 8.2 ± 1.2 log_10_ CFU/g feces, respectively). Phylogenetically, NEC cases and control strains belonged to either phylogroup B2 (25% and 47%, respectively) or phylogroup D (50% and 47%, respectively) (Tables S1 and S2). The strains were heterogeneously distributed among the different capsular groups (Tables S1 and S2). No significant statistical difference was observed between NEC cases and controls in either strain carriage by phylogroup or capsular group (*p* > 0.19).

Overall, of the 54 virulence genes tested, some were either absent (*bmaE*, *rfc*, *papG allele I*, *vat*, *gafD*, *tsh*, *f17*, *clpG*, and *astA*), amplified in at least 50% of the strains (*fyuA*, *kii*, *traT*, *Kps II*, *uidA*, *afaE8*, and *fimH*), or present in all strains (*fimH*) (Tables S1 and S2). Regarding UPEC-associated virulence genes, *papG I*, *papG allele I*, and *papG allele III* were absent in NEC strains, whereas UPEC papG allele I’*, *papG allele II*, *papEF*, *sfa*, and *focG* were amplified in either NEC cases or control strains. Statistically, there was no difference in virulence gene distribution between control and NEC strains (*p* > 0.15) (Tables S1–S3).

All strains were susceptible to imipenem, tigecyclin, and cefoxitin (Table S4). Antimicrobial susceptibility data for beta-lactams showed that the control and NEC strains carried an AmpC beta-lactamase (29% (n = 5) and 25% (n = 1), respectively), extended-spectrum beta-lactamase (ESBL) (35% (n = 6) and 2.5% (n = 1), respectively), or penicillinase (18% (n = 3) and 50% (n = 2), respectively).

### 3.6. C. butyricum and C. neonatale Isolation and Antimicrobial Susceptibility Levels

A total of 33 *C. butyricum* (NEC cases, n = 12; controls, n = 21) and 34 *C. neonatale* strains (NEC cases, n = 9; controls, n = 25) were isolated from the stool samples. By culture, the mean colonization level of *C. butyricum* (5.9 ± 1.7 log_10_ CFU/g feces) and *C. neonatale* (5.0 ± 1.2 log_10_ CFU/g feces) for the NEC cases was similar to the PN controls for both species (6.1 ± 1.4 log_10_ CFU/g feces). *C. butyricum* strains were resistant to amoxicillin (11%) and piperacillin (18%) (Table S5). Based on the nitrocefin assay, all the amoxicillin-resistant strains were positive for β-lactamase activity, which was inhibited by clavulanate. In addition, 4 *C. butyricum* strains were resistant to tetracycline (12%, MICs > 8 mg/L), and 12 to were resistant to cefotaxime (36%, MICs > 32 mg/L) (Table S6). For *C. neonatale*, 21 strains were resistant to clindamycin (62%) (MICs > 4 mg/L), and 4 were resistant to cefotaxime (12%) (Table S6). Statistically, the levels of colonization or antibiotic susceptibility were similar between control and NEC strains (*p* >0.20).

## 4. Discussion

Through this multicenter case–control study, although there were no significant differences in the dominant gut microbiota composition, we showed a gut microbiota dysbiosis between NEC and control infants at the genus level, with a decrease in *Lactobacillus* and an increase in *Clostridium* cluster III.

Regarding the PN gut microbiota, we found that it could be divided into five clusters corresponding to five bacterial enterotypes characterized by either *Enterobacter*, *Clostridium sensu stricto*, *Escherichia*, *Enterococcus*, or *Staphylococcus* dominance. This approach allowed us to summarize the complex bacterial composition into categories, each dominated by these early life-associated taxonomic groups. Of note, the last three clusters were closer together in our cohort. Similar clustering has been described [38], highlighting the high variability of the gut microbiota establishment in the very preterm population. In our PN population, the main phyla detected were Firmicutes and Proteobacteria, in agreement with previous studies [39]. When comparing the gut microbiota of NEC cases and controls, there was no differential clustering of samples. However, the enterotypes dominated by *Escherichia* and *Staphylococcus* had a lower percentage of NEC cases. A hierarchical clustering-based approach defining PN community state types (CSTs) reported by Tarracchini et al. also showed an association of NEC cases with some CSTs [20]. Regarding gut microbiota metrics, we found that the genus *Lactobacillus* was significantly less abundant in NEC cases. The *Lactobacillus* genus is characteristic of the gut microbiota of the gold standard full-term breastfed infants [40], whose colonization is favored by the presence of indigestible oligosaccharides in human milk. Several studies have reported a negative association between antibiotic exposure and *Lactobacillus* abundance [41,42]. In the present study, NEC cases received antibiotic treatment for nosocomial infections more frequently than controls, which may explain our data. We also observed that *Clostridium* cluster III was significantly more represented in NEC cases, although at low proportions. This observation supports previous reports of associations between the *Clostridium* genus and NEC [14,15,16,18,20]. Furthermore, these results were consistent with a dysbiotic status of the gut microbiota and NEC. Data on bacterial abundance at the class or genus level have been proposed, with a wide heterogeneity of results. In fact, some studies have shown no differences between NEC cases and controls [12,13,19,39] or have reported divergent results [7,8,9,10,11,14,15,16,17,43]. As highlighted by previous authors, the differences between studies may be explained by the majority of studies being monocentric with small sample sizes, variability in the population inclusion criteria, NEC definitions, times of sample collection, DNA extraction methods, and metadata analyses. Of note, a review of 17 studies of NEC outbreaks failed to identify a reproducible pattern of bacterial infection [44].

In 2016, based on shotgun metagenomics, UPEC carriage was associated with a significantly higher risk of NEC development and death as an outcome compared to control infants [11]. Although pangenomic approaches allowed for strain resolution by functional subtyping or de novo genome assembly, bacterial culture remains the only method that allows phenotypic characterization of the strains. In the present study, *E. coli* isolates mostly belonged to B2 and D phylogroups, which are associated with the extraintestinal pathogenic *E. coli* (ExPEC) group, including UPEC. Strain carriage was equivalent between NEC cases and controls, in agreement with previous data obtained by culture [45] and different from Ward et al. [11]. These different conclusions may be explained by the methodological differences between the studies. In addition, Ward et al. [11] analyzed samples collected before NEC, whereas our samples were collected at the onset of NEC. As UPEC possesses a diverse repertoire of virulence genes, we screened *E. coli* isolates for ExPEC- and UPEC-specific virulence genes. However, the distribution of virulence genes among the isolates was found to be equivalent between NEC cases and controls.

Preterm infants almost universally receive very early broad-spectrum antibiotics, and many receive prolonged courses of antibiotics for the treatment of culture-proven sepsis or suspected infection [46]. There is increasing evidence of an association between antibiotic use and the abundance of multidrug-resistant bacteria in PNs [47,48]. In addition, although early antibiotic use is associated with a lower incidence of NEC [49], prolonged antenatal and postnatal antibiotic exposure has been shown to be associated with an increased risk of developing NEC [50]. Recently, an association between the colonization frequency of colonization with *Enterobacteriaceae* or *C. butyricum* in patients with NEC and the administration of antibiotics has been proposed [23,24]. Notably, data on the antimicrobial susceptibility of clinical isolates are scarce when clinical treatment of NEC includes broad-spectrum antibiotics, including clindamycin or metronidazole [51]. In the present study, NEC cases received antibiotics more frequently, supporting previous data linking post-natal antibiotic exposure to an increased risk of developing NEC [50]. In addition, the majority of the neonates received antibiotic therapy for neonatal nosocomial infection, including vancomycin or cefotaxime. We found that most of the *E. coli* strains exhibited an enzymatic mechanism involved in beta-lactam resistance (including ESBL production), regardless of strain origin. Previously, a retrospective single-center observational study reported no evidence of ESBL-producing *Enterobacteriaceae* colonization and a higher risk of NEC [52]. We also observed that a few *C. butyricum* and *C. neonatale* strains showed acquired resistance to cefotaxime and that clindamycin or tetracycline resistance was observed in a few *C. neonatale* strains. Recently, two different *C. neonatale* strains were reported to carry the ribosomal protection protein *tet* gene or the 23S rRNA methyltransferase *ermQ* gene [53]. Notably, PNs exposed to antibiotics were reported to be more frequently colonized with *C. butyricum* species [30]. In the present study, although NEC neonates received significantly more antibiotics for nosocomial infections than controls, our results suggested that there was no correlation between antibiotic use and either the level of colonization or the carriage of resistance between the strains isolated from both PN groups.

Although antifungals have been proposed to be directly or indirectly affect the gut bacterial communities, their effects on PN gut microbiota have been largely overlooked [54]. Our data showed that antifungal treatment and lower birth weight or gestational age were associated with significant differences in the microbial compositions of PNs. However, no association was found when comparing NEC cases and controls.

The strength of this study is that it was a multicenter, nationwide matched case–control cohort that allowed the recruitment of a large number of unrelated preterm infants, which allowed us to account for the population variability of the gut microbiota in PNs. In terms of clinical characteristics, the enrolled population was homogeneous, with a careful definition of the cases and matched controls. Another strength is the culture of specific bacteria previously suggested to be associated with NEC, which allowed the determination of strain antimicrobial susceptibility and virulence genes, rarely studied. The description of bacterial composition and diversity in this study is limited by the use of the 16S rRNA gene sequencing approach. Shotgun metagenomic analysis could provide additional information, although the small volume of fecal samples and low bacterial biomass of NEC patients may affect the data. The NICU environment or practices may represent potential perinatal factors influencing the gut microbiota of preterm and very preterm neonates. In the present study, the multicenter characteristics of our population may contribute to explaining the heterogeneity of gut microbiota composition observed within the NEC group. However, the statistical analyses were inconclusive due to low statistical power. Noteworthy, most available NEC studies are monocentric, and the NICU has rarely been investigated as an external perinatal factor. Therefore, large multicenter studies with adequate power are needed to allow investigate the role of the NICU. Another limitation is the single sampling at disease onset. As in previous studies, the accuracy of our results may be limited by confounding factors, particularly concurrent medication.

## 5. Conclusions

Based on a well-characterized multicenter study, we showed that although the dominant gut microbiota composition was similar between groups, there was a dysbiotic state of the gut microbiota at disease onset between PNs with and without NEC. At the strain level, we found an equivalent carriage of UPEC, *C. butyricum*, or *C. neonatale* strains between PN with and without NEC. Studies on bacterial carriage rates and antimicrobial susceptibility in relation to NEC are very limited. Although some strains were phenotypically resistant to some antibiotics, carriage was equivalent between NEC cases and controls. This study also illustrates how the combination of culture and metagenomics methods targeting the bacterial 16S rRNA gene approaches can contribute to a better understanding of the bacterial role in NEC in future studies.

## Figures and Tables

**Table 1:** ClosNEC study patient’s characteristics.

Characteristics	NEC Cases (n = 53)	Controls (n = 93)	*p* Value
**Gestational age (weeks)**; *median (quartiles)*	28 ± 2; *29.0 (26–30)*	28 ± 2; *29.0 (26–30)*	0.85
**Birth weight (g)**; *median (quartiles)*	1077 ± 359; *1000 (820–1280)*	1062 ± 303; *980 (840–1335)*	0.97
**Human milk—no. (%)**	39 (73)	81 (87)	0.54
**Vaginal delivery—no. (%)**	21 (40)	38 (41)	1
**Maternal antibiotic therapy—no. (%)**	24 (45)	39 (41)	0.60
- antenatal	19 (36)	25 (27)	0.26
- per partum	19 (36)	34 (36)	1
**Neonatal antibiotic therapy—no. (%)**	48 (90)	68 (73)	**0.01**
- early therapy *	29 (55)	54 (58)	0.70
duration (days) [min–max]	3 ± 1.5 [1–7]	4 ± 2.2 [1–10]	0.9
- suspicion of maternofeotal or nosocomial infection	45 (83)	48 (52)	**<0.0001**
duration (days) [min–max]	12 ± 6.7 [3–34]	10 ± 8.0 [2–39]	0.07
**Antifungal treatment—no. (%)**	15 (28)	26 (28)	1
duration (days) [min–max]	9 ± 9.6 [1–29]	12 ± 10.0 [1–35]	1

* defined as antibiotic administration initiated at ≤72 h of age.

## Data Availability

The 16S rRNA gene sequencing results are publicly available from the NCBI Sequence Read Archive (SRA) under the Bioproject accession number PRJNA998599. Other data might be available upon reasonable request to the corresponding author.

## Supplementary Materials

The following supporting information can be downloaded at: https://www.mdpi.com/article/10.3390/microorganisms11102457/s1. Table S1. Summary of the 54 virulence genes, phylogroup affiliation, and capsular groups of the 17 *Escherichia coli* strains isolated from controls, as detected by polymerase chain reaction assay; Table S2. Summary of the 54 virulence genes, phylogroup affiliations, and capsular groups of the four *Escherichia coli* strains isolated from NEC cases, as detected by polymerase chain reaction assay; Table S3. Distribution of virulence genes according to the phylogroup B2 and D (*p*-values are calculated using the two-sided Fisher’s exact test; significance: *p* < 0.05); Table S4. Antibiotic susceptibility phenotype of the *Escherichia coli* strains isolated from the controls and NEC cases; Table S5. Antibiotic susceptibility phenotype of the *Clostridium butyricum* strains; Table S6. Minimal inhibitory concentrations (MIC) of *Clostridium butyricum* and *Clostridium neonatale* strains to tetracycline, clindamycin, and cefotaxime; Figure S1. Proportions of bacterial genera significantly different between C-section (CS) and vaginal delivery (VB) preterm neonates. * *p*-value < 0.05.

## References

[1] Heron M. (2018). Deaths: Leading Causes for 2016. Natl. Vital Stat. Rep..

[2] Battersby C., Santhalingam T., Costeloe K., Modi N. (2018). Incidence of Neonatal Necrotising Enterocolitis in High-Income Countries: A Systematic Review. Arch. Dis. Child Fetal Neonatal Ed..

[3] Bazacliu C., Neu J. (2019). Pathophysiology of Necrotizing Enterocolitis: An Update. Curr. Pediatr. Rev..

[4] Neu J. (2020). Necrotizing Enterocolitis: The Future. Neonatology.

[5] Neu J., Walker W.A. (2011). Necrotizing Enterocolitis. N. Engl. J. Med..

[6] Morowitz M.J., Poroyko V., Caplan M., Alverdy J., Liu D.C. (2010). Redefining the Role of Intestinal Microbes in the Pathogenesis of Necrotizing Enterocolitis. Pediatrics.

[7] Wang Y., Hoenig J.D., Malin K.J., Qamar S., Petrof E.O., Sun J., Antonopoulos D.A., Chang E.B., Claud E.C. (2009). 16S RRNA Gene-Based Analysis of Fecal Microbiota from Preterm Infants with and without Necrotizing Enterocolitis. ISME J..

[8] Mai V., Young C.M., Ukhanova M., Wang X., Sun Y., Casella G., Theriaque D., Li N., Sharma R., Hudak M. (2011). Fecal Microbiota in Premature Infants Prior to Necrotizing Enterocolitis. PLoS ONE.

[9] Claud E.C., Keegan K.P., Brulc J.M., Lu L., Bartels D., Glass E., Chang E.B., Meyer F., Antonopoulos D.A. (2013). Bacterial Community Structure and Functional Contributions to Emergence of Health or Necrotizing Enterocolitis in Preterm Infants. Microbiome.

[10] Morrow A.L., Lagomarcino A.J., Schibler K.R., Taft D.H., Yu Z., Wang B., Altaye M., Wagner M., Gevers D., Ward D.V. (2013). Early Microbial and Metabolomic Signatures Predict Later Onset of Necrotizing Enterocolitis in Preterm Infants. Microbiome.

[11] Ward D.V., Scholz M., Zolfo M., Taft D.H., Schibler K.R., Tett A., Segata N., Morrow A.L. (2016). Metagenomic Sequencing with Strain-Level Resolution Implicates Uropathogenic *E. coli* in Necrotizing Enterocolitis and Mortality in Preterm Infants. Cell Rep..

[12] Normann E., Fahlén A., Engstrand L., Lilja H.E. (2013). Intestinal Microbial Profiles in Extremely Preterm Infants with and without Necrotizing Enterocolitis. Acta Paediatr..

[13] Raveh-Sadka T., Thomas B.C., Singh A., Firek B., Brooks B., Castelle C.J., Sharon I., Baker R., Good M., Morowitz M.J. (2015). Gut Bacteria Are Rarely Shared by Co-Hospitalized Premature Infants, Regardless of Necrotizing Enterocolitis Development. Elife.

[14] Cassir N., Benamar S., Khalil J.B., Croce O., Saint-Faust M., Jacquot A., Million M., Azza S., Armstrong N., Henry M. (2015). *Clostridium butyricum* Strains and Dysbiosis Linked to Necrotizing Enterocolitis in Preterm Neonates. Clin. Infect. Dis..

[15] Roze J.C., Ancel P.Y., Lepage P., Martin-Marchand L., Al N.Z., Delannoy J., Picaud J.C., Lapillonne A., Aires J., Durox M. (2017). Nutritional Strategies and Gut Microbiota Composition as Risk Factors for Necrotizing Enterocolitis in Very-Preterm Infants. Am. J. Clin. Nutr..

[16] Sim K., Shaw A.G., Randell P., Cox M.J., McClure Z.E., Li M.S., Haddad M., Langford P.R., Cookson W.O., Moffatt M.F. (2015). Dysbiosis Anticipating Necrotizing Enterocolitis in Very Premature Infants. Clin. Infect. Dis..

[17] Zhou Y., Shan G., Sodergren E., Weinstock G., Walker W.A., Gregory K.E. (2015). Longitudinal Analysis of the Premature Infant Intestinal Microbiome Prior to Necrotizing Enterocolitis: A Case-Control Study. PLoS ONE.

[18] Coggins S.A., Wynn J.L., Weitkamp J.H. (2015). Infectious Causes of Necrotizing Enterocolitis. Clin. Perinatol..

[19] Pammi M., Cope J., Tarr P.I., Warner B.B., Morrow A.L., Mai V., Gregory K.E., Kroll J.S., McMurtry V., Ferris M.J. (2017). Intestinal Dysbiosis in Preterm Infants Preceding Necrotizing Enterocolitis: A Systematic Review and Meta-Analysis. Microbiome.

[20] Tarracchini C., Milani C., Longhi G., Fontana F., Mancabelli L., Pintus R., Lugli G.A., Alessandri G., Anzalone R., Viappiani A. (2021). Unraveling the Microbiome of Necrotizing Enterocolitis: Insights in Novel Microbial and Metabolomic Biomarkers. Microbiol. Spectr..

[21] Bernard K., Burdz T., Wiebe D., Alfa M., Bernier A.-M. (2018). *Clostridium neonatale* Sp. Nov. Linked to Necrotizing Enterocolitis in Neonates and a Clarification of Species Assignable to the Genus *Clostridium* (Prazmowski 1880) Emend. Lawson and Rainey 2016. Int. J. Syst. Evol. Microbiol..

[22] Cassir N., Grandvuillemin I., Boxberger M., Jardot P., Boubred F., La Scola B. (2021). Case Report: *Clostridium neonatale* Bacteremia in a Preterm Neonate With Necrotizing Enterocolitis. Front. Pediatr..

[23] Hosny M., Bou Khalil J.Y., Caputo A., Abdallah R.A., Levasseur A., Colson P., Cassir N., La Scola B. (2019). Multidisciplinary Evaluation of *Clostridium butyricum* Clonality Isolated from Preterm Neonates with Necrotizing Enterocolitis in South France between 2009 and 2017. Sci. Rep..

[24] Olm M.R., Bhattacharya N., Crits-Christoph A., Firek B.A., Baker R., Song Y.S., Morowitz M.J., Banfield J.F. (2019). Necrotizing Enterocolitis Is Preceded by Increased Gut Bacterial Replication, *Klebsiella*, and Fimbriae-Encoding Bacteria. Sci. Adv..

[25] Lepage P., Seksik P., Sutren M., de la Cochetiere M.F., Jian R., Marteau P., Dore J. (2005). Biodiversity of the Mucosa-Associated Microbiota Is Stable along the Distal Digestive Tract in Healthy Individuals and Patients with IBD. Inflamm. Bowel. Dis..

[26] Furet J.P., Firmesse O., Gourmelon M., Bridonneau C., Tap J., Mondot S., Dore J., Corthier G. (2009). Comparative Assessment of Human and Farm Animal Faecal Microbiota Using Real-Time Quantitative PCR. FEMS Microbiol. Ecol..

[27] Wilson K.H., Blitchington R.B., Greene R.C. (1990). Amplification of Bacterial 16S Ribosomal DNA with Polymerase Chain Reaction. J. Clin. Microbiol..

[28] Caporaso J.G., Kuczynski J., Stombaugh J., Bittinger K., Bushman F.D., Costello E.K., Fierer N., Pena A.G., Goodrich J.K., Gordon J.I. (2010). QIIME Allows Analysis of High-Throughput Community Sequencing Data. Nat. Methods.

[29] Cole J.R., Wang Q., Cardenas E., Fish J., Chai B., Farris R.J., Kulam-Syed-Mohideen A.S., McGarrell D.M., Marsh T., Garrity G.M. (2009). The Ribosomal Database Project: Improved Alignments and New Tools for RRNA Analysis. Nucleic Acids Res..

[30] Ferraris L., Butel M.J., Campeotto F., Vodovar M., Roze J.C., Aires J. (2012). Clostridia in Premature Neonates’ Gut: Incidence, Antibiotic Susceptibility, and Perinatal Determinants Influencing Colonization. PLoS ONE.

[31] Bouvet P., Ferraris L., Dauphin B., Popoff M.R., Butel M.J., Aires J. (2014). 16S RRNA Gene Sequencing, Multilocus Sequence Analysis, and Mass Spectrometry Identification of the Proposed New Species “Clostridium neonatale”. J. Clin. Microbiol..

[32] Clermont O., Christenson J.K., Denamur E., Gordon D.M. (2013). The Clermont *Escherichia coli* Phylo-Typing Method Revisited: Improvement of Specificity and Detection of New Phylo-Groups. Environ. Microbiol. Rep..

[33] Chapman T.A., Wu X.Y., Barchia I., Bettelheim K.A., Driesen S., Trott D., Wilson M., Chin J.J. (2006). Comparison of Virulence Gene Profiles of *Escherichia coli* Strains Isolated from Healthy and Diarrheic Swine. Appl. Environ. Microbiol..

[34] Johnson J.R., O’Bryan T.T. (2004). Detection of the *Escherichia coli* Group 2 Polysaccharide Capsule Synthesis Gene *kpsM* by a Rapid and Specific PCR-Based Assay. J. Clin. Microbiol..

[35] The European Committee on Antimicrobial Susceptibility Testing (2023). Breakpoint Tables for Interpretation of MICs and Zone Diameters, Version 13.0. http://www.eucast.org.

[36] Benjamini Y., Drai D., Elmer G., Kafkafi N., Golani I. (2001). Controlling the False Discovery Rate in Behavior Genetics Research. Behav. Brain Res..

[37] Arumugam M., Raes J., Pelletier E., Le P.D., Yamada T., Mende D.R., Fernandes G.R., Tap J., Bruls T., Batto J.M. (2011). Enterotypes of the Human Gut Microbiome. Nature.

[38] Cuna A., Morowitz M.J., Ahmed I., Umar S., Sampath V. (2021). Dynamics of the Preterm Gut Microbiome in Health and Disease. Am. J. Physiol. Gastrointest. Liver Physiol..

[39] Lee J.K.-F., Hern Tan L.T., Ramadas A., Ab Mutalib N.-S., Lee L.-H. (2020). Exploring the Role of Gut Bacteria in Health and Disease in Preterm Neonates. Int. J. Environ. Res. Public Health.

[40] Fouhy F., Guinane C.M., Hussey S., Wall R., Ryan C.A., Dempsey E.M., Murphy B., Ross R.P., Fitzgerald G.F., Stanton C. (2012). High-Throughput Sequencing Reveals the Incomplete, Short-Term Recovery of Infant Gut Microbiota Following Parenteral Antibiotic Treatment with Ampicillin and Gentamicin. Antimicrob. Agents Chemother..

[41] Korpela K., Salonen A., Virta L.J., Kekkonen R.A., De Vos W.M. (2016). Association of Early-Life Antibiotic Use and Protective Effects of Breastfeeding: Role of the Intestinal Microbiota. JAMA Pediatr..

[42] Esaiassen E., Hjerde E., Cavanagh J.P., Pedersen T., Andresen J.H., Rettedal S.I., Støen R., Nakstad B., Willassen N.P., Klingenberg C. (2018). Effects of Probiotic Supplementation on the Gut Microbiota and Antibiotic Resistome Development in Preterm Infants. Front. Pediatr..

[43] Mshvildadze M., Neu J., Shuster J., Theriaque D., Li N., Mai V. (2010). Intestinal Microbial Ecology in Premature Infants Assessed with Non-Culture-Based Techniques. J. Pediatr..

[44] Boccia D., Stolfi I., Lana S., Moro M.L. (2001). Nosocomial Necrotising Enterocolitis Outbreaks: Epidemiology and Control Measures. Eur. J. Pediatr..

[45] Hoy C.M., Wood C.M., Hawkey P.M., Puntis J.W.L. (2000). Duodenal Microflora in Very-Low-Birth-Weight Neonates and Relation to Necrotizing Enterocolitis. J. Clin. Microbiol..

[46] Dierikx T.H., Deianova N., Groen J., Vijlbrief D.C., Hulzebos C., de Boode W.P., d’Haens E.J., Cossey V., Kramer B.W., van Weissenbruch M.M. (2022). Association between Duration of Early Empiric Antibiotics and Necrotizing Enterocolitis and Late-Onset Sepsis in Preterm Infants: A Multicenter Cohort Study. Eur. J. Pediatr..

[47] Gibson M.K., Wang B., Ahmadi S., Burnham C.-A.D., Tarr P.I., Warner B.B., Dantas G. (2016). Developmental Dynamics of the Preterm Infant Gut Microbiota and Antibiotic Resistome. Nat. Microbiol..

[48] Greenwood C., Morrow A.L., Lagomarcino A.J., Altaye M., Taft D.H., Yu Z., Newburg D.S., Ward D.V., Schibler K.R. (2014). Early Empiric Antibiotic Use in Preterm Infants Is Associated with Lower Bacterial Diversity and Higher Relative Abundance of *Enterobacter*. J. Pediatr..

[49] Li Y., Shen R.L., Ayede A.I., Berrington J., Bloomfield F.H., Busari O.O., Cormack B.E., Embleton N.D., van Goudoever J.B., Greisen G. (2020). Early Use of Antibiotics Is Associated with a Lower Incidence of Necrotizing Enterocolitis in Preterm, Very Low Birth Weight Infants: The NEOMUNE-NeoNutriNet Cohort Study. J. Pediatr..

[50] Silverman M.A., Konnikova L., Gerber J.S. (2017). Impact of Antibiotics on Necrotizing Enterocolitis and Antibiotic-Associated Diarrhea. Gastroenterol. Clin. N. Am..

[51] Gill E.M., Jung K., Qvist N., Ellebaek M.B. (2022). Antibiotics in the Medical and Surgical Treatment of Necrotizing Enterocolitis. A Systematic Review. BMC Pediatr..

[52] Eberhart M., Grisold A., Lavorato M., Resch E., Trobisch A., Resch B. (2020). Extended-Spectrum Beta-Lactamase (ESBL) Producing Enterobacterales in Stool Surveillance Cultures of Preterm Infants Are No Risk Factor for Necrotizing Enterocolitis: A Retrospective Case-Control Study over 12 Years. Infection.

[53] Mesa V., Monot M., Ferraris L., Popoff M., Mazuet C., Barbut F., Delannoy J., Dupuy B., Butel M.J., Aires J. (2022). Core-, Pan- and Accessory Genome Analyses of *Clostridium neonatale*: Insights into Genetic Diversity. Microb. Genom..

[54] Wheeler M.L., Limon J.J., Bar A.S., Leal C.A., Gargus M., Tang J., Brown J., Funari V.A., Wang H.L., Crother T.R. (2016). Immunological Consequences of Intestinal Fungal Dysbiosis. Cell Host Microbe.

