# Supplementary material for: Occurrence of Neonatal Necrotizing Enterocolitis in Premature Neonates and Gut Microbiota: A Case–Control Prospective Multicenter Study
Source: Microorganisms. 2023 Sep 29;11(10):2457. doi: 10.3390/microorganisms11102457 (PMC10609581; doi:10.3390/microorganisms11102457)

**Table S1.** Summary of the 54 virulence genes, phylogroup affiliation, and capsular groups of the *Escherichia coli* strains isolated from controls, as detected by polymerase chain reaction assay.

| Strain ID | 311 | 608 | C277 | 606 | C276 | C469 | 561 | 623 | 306 | C7 | C8 | C9 | C79 | C80 | C196 | C206 | C233 |
|---|---|---|---|---|---|---|---|---|---|---|---|---|---|---|---|---|---|
| Phylogroup | A | B2 | B2 | B2 | B2 | B2 | B2 | B2 | B2 | D | D | D | D | D | D | D | D |
| Capsular group | *NA* | K1 | K1/K5 | K5 | K5 | K2/K100 | *NA* | *NA* | *NA* | K1 | K1 | K1 | K15 | K15 | Group 3 | Group 3 | Group 3 |
| *maIX (PAI)* | | | • | | • | • | | | | | | | | | | | |
| *K15* | | | | | | | | | | | | | • | • | | | |
| *fimH* | • | • | • | • | • | • | • | • | • | • | • | • | • | • | • | • | • |
| *kpslll* | | | | | | | | | | | | | | | • | • | • |
| *yfcV* | | • | • | • | • | • | • | | • | | | | | | | | |
| *ibeA* | | | | | | | | • | | | | | | | | | |
| *concnf* | | | • | • | • | | | | | | | | | | | | |
| *fyuA* | | • | • | • | • | • | • | | • | | | | • | • | | • | • |
| *clbB* | | • | • | • | • | • | • | | | • | • | • | | | | • | • |
| *bmaE* | | | | | | | | | | | | | | | | | |
| *iutA* | | | • | • | • | • | • | | • | | | | | | • | • | |
| *hra* | | | | • | | | | | | | | | | • | | | |
| *hlyD* | | | | • | | | • | | | | | | | | | | |
| *rfc* | | | | | | | | | | | | | | | | | |
| *ompT* | | • | • | • | • | • | • | • | • | | • | • | | | | | • |
| *iss* | | | | | | | | | • | | | | | | | | |
| *kii* | | • | • | • | • | | • | | • | • | • | • | | | | | |
| *vat* | | | | | | | | | | | | | | | | | |
| *gafD* | | | | | | | | | | | | | | | | | |
| *K5* | | | • | | • | | | | | | | | | | | | |
| *cvaC* | | | | | | | | | • | | | | | | | | |
| *H7* | | | | | | | | | | | | | | | | | |
| *cdtB/cdtB'* | | | • | | • | | | | | | | | | | | | |
| *traT* | | • | • | | • | • | • | | • | • | • | • | • | • | | | • |
| *iha* | | | • | • | • | • | • | | | | | | | | | • | • |

| | | | | | | | | | | | | | | | | | |
|---|---|---|---|---|---|---|---|---|---|---|---|---|---|---|---|---|---|
| *hlyF* | | | | | | | | | ● | | | | | | | | ● |
| *tsh* | | | | | | | | | | | | | | | | | |
| *sfaS* | | | | | | ● | | | | | | | | | | | |
| *K1* | | ● | ● | | | | | | ● | ● | ● | ● | | | | | |
| *sat* | | | ● | ● | ● | ● | ● | | | | | | | | | | |
| *clbN* | | ● | ● | ● | ● | ● | ● | | | | | | | | | | |
| *f17* | | | | | | | | | | | | | | | | | |
| *kpsII* | | ● | ● | ● | ● | ● | ● | | ● | ● | ● | ● | | | | | |
| *uidA* | ● | ● | ● | ● | ● | ● | ● | ● | ● | ● | ● | ● | ● | ● | ● | ● | ● |
| *usp* | | ● | ● | ● | ● | ● | ● | | ● | | | | | | | | |
| *clpG* | | | | | | | | | | | | | | | | | |
| *afaE8* | | ● | ● | ● | ● | ● | ● | ● | ● | ● | ● | ● | ● | ● | ● | ● | ● |
| *chuA* | | | | | | | | | | | | | | | | | |
| *astA (EAST)* | | | | | | | | | | | | | | | | | |
| *papG I* | | | | | | ● | | | | | | | | | | | |
| *papG II, III* | | ● | ● | ● | ● | | ● | | | | | | | | | | ● |
| *papG allele I* | | | | | | | | | | | | | | | | | |
| *papG allele I'** | | | | | | | | | | | ● | ● | | | ● | ● | ● |
| *papG allele II* | | ● | ● | ● | ● | | ● | | | | | | | | | | |
| *papG allele III* | | | | | | ● | | | | | | | | | | | |
| *papAH* | | | ● | | ● | ● | | | | | | | ● | ● | | | |
| *papC* | | ● | ● | ● | ● | ● | ● | | | | | | ● | ● | | | |
| *papEF* | | | ● | | ● | ● | | | | | | | | | | | |
| *afa* | | | | | | | | | | | | | | | | ● | ● |
| *sfa* | | | ● | ● | ● | ● | | | | | | | | | | | |
| *focG* | | | ● | ● | ● | ● | | | | | | | | | | | |
| *pic* | | | | | | | | | | | | | | | | | |
| *ireA* | | ● | ● | ● | ● | | ● | | | | | | | | | | |
| *iroNec* | | | ● | ● | ● | ● | ● | | ● | ● | | | | | | | |

●: positive results. *NA*: unidentified.

**Table S2.** Summary of the 54 virulence genes, phylogroup affiliation, and capsular groups of the *Escherichia coli* strains isolated from NEC cases, as detected by polymerase chain reaction assay.

| Strain ID | C439 | C74 | C345 | C325 |
|---|---|---|---|---|
| Phylogroup | B2 | D | D | E |
| Capsular group | *NA* | K15 | Group 3 | K1 |
| *maIX (PAI)* | ● | | | |
| *K15* | | ● | | |
| *fimH* | ● | ● | ● | ● |
| *kpslll* | | | ● | |
| *yfcV* | ● | | | |
| *ibeA* | | | | |
| *concnf* | | | | |
| *fyuA* | | ● | ● | |
| *clbB* | ● | | | |
| *bmaE* | | | | |
| *iutA* | | | | ● |
| *hra* | | | | |
| *hlyD* | | | | |
| *rfc* | | | | |
| *ompT* | | | | ● |
| *iss* | | | | |
| *kii* | | | | ● |
| *vat* | | | | |
| *gafD* | | | | |
| *K5* | | | | |
| *cvaC* | | | | ● |
| *H7* | | | | |
| *cdtB/cdtB'* | | | | ● |
| *traT* | | | ● | ● |
| *iha* | ● | ● | | |
| *hlyF* | | | | ● |

| | | | | |
|---|---|---|---|---|
| *tsh* | | | | |
| *sfaS* | | | | |
| *K1* | | | | |
| *sat* | | | | |
| *clbN* | | | | |
| *f17* | | | | |
| *kpsII* | | | | ● |
| *uidA* | ● | ● | ● | ● |
| *usp* | | | | ● |
| *clpG* | | | | |
| *afaE8* | ● | | ● | ● |
| *chuA* | | | | |
| *astA (EAST)* | | | | |
| *papG I* | | | | |
| *papG II, III* | ● | | ● | |
| *papG allele I* | | | | |
| *papG allele I'** | | | | ● |
| *papG allele II* | | | | |
| *papG allele III* | | | | |
| *papAH* | | ● | | |
| *papC* | | | ● | |
| *papEF* | | | | |
| *afa* | ● | | ● | |
| *sfa* | | | | |
| *focG* | | | | |
| *pic* | | | | |
| *ireA* | | | | |
| *iroNec* | | | | ● |

●: positive results. *NA*: unidentified.

**Table S3.** Distribution of virulence genes according to the phylogroup B2 and D (p-values are calculated using the two-sided Fisher's exact test; significance: p<0.05).

| Virulence genes | Phylogroup B2 | | | Phylogroup D | | |
|---|---|---|---|---|---|---|
| | Control (n = 8) % (n) | NEC (n = 1) % (n) | *p* | Control (n = 8) % (n) | NEC (n = 2) % (n) | *p* |
| *malX (PAI)* | 37,5 (3) | 100 (1) | 0.44 | 0 (0) | 0 (0) | 1.00 |
| *K15* | 0 (0) | 0 (0) | 1.00 | 25 (2) | 50 (1) | 1.00 |
| *fimH* | 100 (8) | 100 (1) | 1.00 | 100 (8) | 100 (2) | 1.00 |
| *kpsIII* | 0 (0) | 0 (0) | 1.00 | 37,5 (3) | 50 (1) | 1.00 |
| *yfcV* | 87,5 (7) | 100 (1) | 1.00 | 0 (0) | 0 (0) | 1.00 |
| *ibeA* | 12,5 (1) | 0 (0) | 1.00 | 0 (0) | 0 (0) | 1.00 |
| *concnf* | 37,5 (3) | 0 (0) | 1.00 | 0 (0) | 0 (0) | 1.00 |
| *fyuA* | 87,5 (7) | 0 (0) | 0.22 | 50 (4) | 100 (2) | 0.47 |
| *clbB* | 75 (6) | 100 (1) | 1.00 | 62,5 (5) | 0 (0) | 0.44 |
| *bmaE* | 0 (0) | 0 (0) | 1.00 | 0 (0) | 0 (0) | 1.00 |
| *iutA* | 75 (6) | 0 (0) | 0.33 | 25 (2) | 0 (0) | 1.00 |
| *hra* | 12,5 (1) | 0 (0) | 1.00 | 12,5 (1) | 0 (0) | 1.00 |
| *hlyD* | 25 (2) | 0 (0) | 1.00 | 0 (0) | 0 (0) | 1.00 |
| *rfc* | 0 (0) | 0 (0) | 1.00 | 0 (0) | 0 (0) | 1.00 |
| *ompt* | 100 (8) | 0 (0) | 0.11 | 37,5 (3) | 0 (0) | 1.00 |
| *iss* | 12,5 (1) | 0 (0) | 1.00 | 0 (0) | 0 (0) | 1.00 |
| *kii* | 75 (6) | 0 (0) | 0.33 | 37,5 (3) | 0 (0) | 1.00 |
| *vat* | 0 (0) | 0 (0) | 1.00 | 0 (0) | 0 (0) | 1.00 |
| *gafD* | 0 (0) | 0 (0) | 1.00 | 0 (0) | 0 (0) | 1.00 |
| *K5* | 25 (2) | 0 (0) | 1.00 | 0 (0) | 0 (0) | 1.00 |
| *cvaC* | 12,5 (1) | 0 (0) | 1.00 | 0 (0) | 0 (0) | 1.00 |
| *H7* | 0 (0) | 0 (0) | 1.00 | 0 (0) | 0 (0) | 1.00 |
| *cdtB/cdtB'* | 25 (2) | 0 (0) | 1.00 | 0 (0) | 0 (0) | 1.00 |
| *traT* | 75 (6) | 0 (0) | 0.33 | 75 (6) | 50 (1) | 1.00 |
| *iha* | 62,5 (5) | 100 (1) | 1.00 | 25 (2) | 50 (1) | 1.00 |
| *hlyF* | 12,5 (1) | 0 (0) | 1.00 | 12,5 (1) | 0 (0) | 1.00 |
| *tsh* | 0 (0) | 0 (0) | 1.00 | 0 (0) | 0 (0) | 1.00 |
| *sfaS* | 12,5 (1) | 0 (0) | 1.00 | 0 (0) | 0 (0) | 1.00 |
| *K1* | 37,5 (3) | 0 (0) | 1.00 | 37,5 (3) | 0 (0) | 1.00 |
| *sat* | 62,5 (5) | 0 (0) | 0.44 | 0 (0) | 0 (0) | 1.00 |
| *clbN* | 75 (6) | 0 (0) | 0.33 | 0 (0) | 0 (0) | 1.00 |
| *f17* | 0 (0) | 0 (0) | 1.00 | 0 (0) | 0 (0) | 1.00 |
| *kipsII* | 87,5 (7) | 0 (0) | 0.22 | 37,5 (3) | 0 (0) | 1.00 |
| *uidA* | 100 (8) | 100 (1) | 1.00 | 100 (8) | 100 (2) | 1.00 |
| *usp* | 87,5 (7) | 0 (0) | 0.22 | 0 (0) | 0 (0) | 1.00 |
| *clpG* | 0 (0) | 0 (0) | 1.00 | 0 (0) | 0 (0) | 1.00 |
| *afaE8* | 100 (8) | 100 (1) | 1.00 | 100 (8) | 50 (1) | 0.20 |
| *chuA* | 0 (0) | 0 (0) | 1.00 | 0 (0) | 0 (0) | 1.00 |

| | | | | | | |
|---|---|---|---|---|---|---|
| *astA (EAST)* | 0 (0) | 0 (0) | 1.00 | 0 (0) | 0 (0) | 1.00 |
| *papG I* | 12,5 (1) | 0 (0) | 1.00 | 0 (0) | 0 (0) | 1.00 |
| *papG II, III* | 62,5 (5) | 100 (1) | 1.00 | 12,5 (1) | 50 (1) | 0.38 |
| *papG allele I* | 0 (0) | 0 (0) | 1.00 | 0 (0) | 0 (0) | 1.00 |
| *papG allele I'** | 0 (0) | 0 (0) | 1.00 | 62,5 (5) | 0 (0) | 0.44 |
| *papG allele II* | 62,5 (5) | 0 (0) | 0.44 | 0 (0) | 0 (0) | 1.00 |
| *papG allele III* | 12,5 (1) | 0 (0) | 1.00 | 0 (0) | 0 (0) | 1.00 |
| *papAH* | 37,5 (3) | 0 (0) | 1.00 | 25 (2) | 50 (1) | 1.00 |
| *papC* | 75 (6) | 0 (0) | 0.33 | 25 (2) | 50 (1) | 1.00 |
| *papEF* | 37,5 (3) | 0 (0) | 1.00 | 0 (0) | 0 (0) | 1.00 |
| *afa* | 0 (0) | 100 (1) | 0.11 | 25 (2) | 50 (1) | 1.00 |
| *sfa* | 50 (4) | 0 (0) | 1.00 | 0 (0) | 0 (0) | 1.00 |
| *focG* | 50 (4) | 0 (0) | 1.00 | 0 (0) | 0 (0) | 1.00 |
| *pic* | 0 (0) | 0 (0) | 1.00 | 0 (0) | 0 (0) | 1.00 |
| *ireA* | 62,5 (5) | 0 (0) | 0.44 | 0 (0) | 0 (0) | 1.00 |
| *iroNec* | 75 (6) | 0 (0) | 0.33 | 12,5 (1) | 0 (0) | 1.00 |

**Table S4.** Antimicrobial susceptibility phenotype of the *Escherichia coli* strains isolated from the controls and the NEC cases.

**Controls**

| Antibiotics | % (n) | | |
|---|---|---|---|
| | S* | I | R |
| Amoxicillin | 47 (8) | | 53 (9) |
| Amoxicillin + Clavulanic acid | 59 (10) | | 41 (7) |
| Ticarcillin | 47 (8) | | 53 (9) |
| Ticarcillin + Clavulanic acid | 52 (9) | 24 (4) | 24 (4) |
| Piperacillin + Tazobactam | 41 (7) | | 59 (10) |
| Cefepim | 52 (9) | 24 (4) | 24 (4) |
| Cefotaxim | 65 (11) | | 35 (6) |
| Cefoxitin | 100 (17) | | |
| Imipenem | 100 (17) | | |
| Nalidixic acid | 88 (15) | | 12 (2) |
| Ciprofloxacin | 71 (12) | | 29 (5) |
| Ofloxacin | 47 (8) | 18 (3) | 35 (6) |
| Amikacin | 65 (11) | | 35 (6) |
| Gentamicin | 65 (11) | | 35 (6) |
| Tigecyclin | 100 (17) | | |
| Trimethoprim + Sulfamethoxazol | 76 (13) | | 24 (4) |

**NEC cases**

| Antibiotics | % (n) | | |
|---|---|---|---|
| | S* | I | R |
| Amoxicillin | 25 (1) | | 75 (3) |
| Amoxicillin + Clavulanic acid | 25 (1) | | 75 (3) |
| Ticarcillin | 25 (1) | | 75 (3) |
| Ticarcillin + Clavulanic acid | 25 (1) | | 75 (3) |
| Piperacillin + Tazobactam | 25 (1) | | 75 (3) |
| Cefepim | 25 (1) | 50 (2) | 25 (1) |
| Cefotaxim | 50 (2) | | 50 (2) |

| | | | |
|---|---|---|---|
| Cefoxitin | 100 (4) | | |
| Imipenem | 100 (4) | | |
| Nalidixic acid | 75 (3) | | 25 (1) |
| Ciprofloxacin | 100 (4) | | |
| Ofloxacin | 25 (1) | 50 (2) | 25 (1) |
| Amikacin | 50 (2) | | 50 (2) |
| Gentamicin | 75 (3) | | 25 (1) |
| Tigecyclin | 100 (4) | | |
| Trimethoprim + Sulfamethoxazol | 75 (3) | | 25 (1) |

* S, susceptible; I, intermediate; R, resistant [39].

**Table S5.** Antimicrobial susceptibility phenotype of the *Clostridium butyricum* strains.

| | % (n) | | |
|---|---|---|---|
| Antibiotics | S* | I | R |
| Amoxicillin | 85 (28) | 3 (1) | 11 (4) |
| Amoxicillin/clavulanic acid | 100 (33) | | |
| Piperacillin | 82 (27) | | 18 (6) |
| Piperacillin/tazobactam | 88 (29) | 12 (4) | |
| Cefoxitin | 100 (33) | | |
| Ertapenem | 100 (33) | | |
| Imipenem | 100 (33) | | |
| Chloramphenicol | 100 (33) | | |
| Linezolide | 100 (33) | | |
| Metronidazol | 100 (33) | | |
| Moxifloxacin | 67 (22) | 33 (11) | |
| Tigecyclin | 61 (20) | | 39 (13) |
| Vancomycine | 100 (33) | | |

* S, susceptible; I, intermediate; R, resistant [39].

**Table S6.** Minimal inhibitory concentrations (MICs) of *Clostridium butyricum* and *Clostridium neonatale* strains to tetracycline, clindamycin, and cefotaxime.

| | no. of strains for which the antimicrobial agent MIC (mg/L) was: | | | | | | | | | | | | | | | MIC (mg/L) | |
|---|---|---|---|---|---|---|---|---|---|---|---|---|---|---|---|---|---|
| | ≤0.19 | 0.25 | 0.38 | 0.5 | 1 | 1.5 | 2 | 3 | 4 | 6 | 8 | 12 | 16 | 32 | >32 | MIC 50% | MIC 90% |
| *Clostridium butyricum* | | | | | | | | | | | | | | | | | |
| Tetracycline | 17 | | | | | | 2 | 5 | 2 | 2 | 1 | **1** | **2** | | **1** | 0.19 | 12 |
| Clindamycin | 28 | | 2 | 1 | | | 1 | | 1 | | | | | | | 0.125 | 0.38 |
| Cefotaxime | 2 | 4 | 3 | | 1 | | | | | | | 1 | | 10 | **12** | 32 | >32 |
| *Clostridium neonatale* | | | | | | | | | | | | | | | | | |
| Tetracycline | 8 | | | | 2 | 7 | 11 | 2 | 2 | 2 | | | | | | 1.5 | 4 |
| Clindamycin | 1 | | | | | | 4 | 2 | 6 | 9 | 5 | 5 | **2** | | | 6 | 12 |
| Cefotaxime | | | | | | | | | | | 1 | | 7 | 22 | **4** | 32 | >32 |

Resistance definition: clindamycin MICs > 4 mg/L; cefotaxime MICs > 32 mg/L; tetracycline MICs > 8 mg/L [39].

**Figure S1.** Proportions of bacterial genera significantly different between C-section (CS) and vaginal delivery (VB) preterm neonates. * *p*-value <0.05.

Mis en forme : Police :Italique

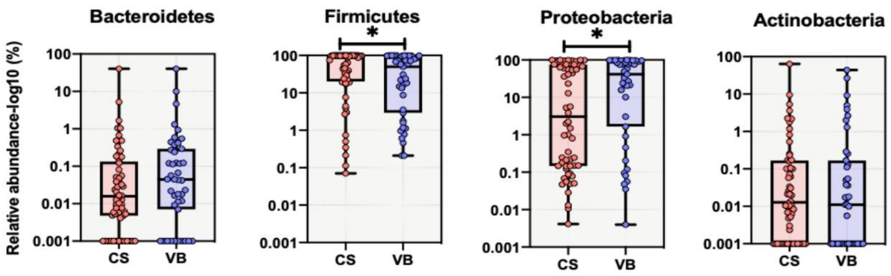

Supplement: Supplementary file 1 [file microorganisms-11-02457-s001.zip › microorganisms-2637299-supplementary.pdf]
